# Brain perfusion permutations during migraine attacks without aura

**DOI:** 10.1186/1129-2377-14-S1-P98

**Published:** 2013-02-21

**Authors:** I Koreshkina, D Khalikov, V Nazinkina, V Amelin, V Osipova

**Affiliations:** 1 Ltd AVA-PETER, Russian Federation; 2 Saint Petersburg State Medical Academy named after I. I. Mechnikov, Russian Federation; 3 Laboratory of Neurovisualization, Institute of the Human Brain of the Russian Academy of Sciences, Russian Federation; 4 Neurology Department, Saint Petersburg State Medical University named after Academician I. P. Pavlov, Russian Federation; 5 Department of Neurology and Clinical Neurophysiology, First Moscow Medical University named after I. M. Sechenov; General Secret, Russian Federation

## Introduction

Migraine attacks are caused by a CNS dysfunction, that activates trigemino-vascular system and is subsequently followed by neurogenic inflammation, dilatation of the cerebral blood vessels, and headache In patients with migraine with aura it has been demonstrated that vascular dilatation at the height of a migraine attack is accompanied by cerebral hyperperfusion According to the majority of studies, cerebral hemodynamic changes are homolateral to the unilateral headache side, and may be confined either to the frontal “ temporal“ parietal region.

## Objective

The main goal of our study was to gain knowledge on blood supply changes during an attack in patients with migraine without aura using contrast-enhanced perfusion-weighted MRI (PWI). Design Standard MRI, and PWI were performed twice: during an attack (in all three cases) and in between attacks (in two cases). Three females suffering from migraine without aura, as per the ICHD-2 diagnostic criteria, underwent brain MRI during a headache attack. Interventions Contrast medium (Magnevist 20 mL) was administered intravenously.

## Results

MRI was performed on a 3.0T Signa HDx Scanner (GE). The study included standard MRI protocol (Ð¢1, Ð¢2, and FLAIR) for assessment of the brain structures, as well as perfusion-weighted MRI (PWI). Cerebral blood volume (CBV), mean transit time of the contrast bolus (MTT), and cerebral blood flow (CBF) were calculated using Functool package. All three cases produced similar local MR perfusion changes that included reduced CBV and decreased CBF without any change in MTT.As the application software did not allow assessment of absolute perfusion values, all obtained results were compared with those for the symmetrical region of the contralateral hemisphere.

## Conclusions

Cerebral blood supply decrease observed during migraine attack is reversible and absent during headache-free interval. Transient perfusion abnormalities occurring during recurrent attacks and confirmed by PWI may serve as one of the mechanisms causing the small subcortical white matter lesions.
